# Progress in Neurology

**Published:** 1901-01-12

**Authors:** 


### PROGRESS IN NEUROLOGY.

(*Continued from page* 210.)

**Hemiplegia.**—Ferrier,[5] at the thirteenth Congress of Medicine, discussed the differential diagnosis of organic and hysterical hemiplegia. He said there was scarcely a single symptom uniformly present sufficient in itself to differentiate the one from the other. A correct diagnosis was, however, in most cases possible, and this depended on a consideration of the personal and family history, the mode of onset, the characters of the paralysis, the course and termination, and the state of the superficial and deep reflexes. The subjects of hysterical hemiplegia were often of neurotic inheritance, who had been previously liable to hysterical attacks or exhibited some of the permanent stigmata of hysteria, such as zones of hyperæsthesia or anæsthesia, or complete sensitivo-sensorial hemianæsthesia of the recognised type. The subjects of organic hemiplegia were those predisposed by vascular, renal, or cardiac disease to cerebral hæmorrhage, embolism, or thrombosis. Hysterical hemiplegia occurred most commonly after some nervous disturbance, such as emotional shock, or after an epileptiform or apoplectiform attack (so-called hysterical apoplexy) simulating cerebral hæmorrhage. In hysterical hemiplegia the upper or lower extremity might be more or less completely powerless and flaccid, but the face was rarely so. Such affection of the face as occurred was usually of the labio-glossal spasmodic type on the same or the opposite side. The leg was usually more affected than the arm, and in walking the paralysed leg was dragged like an inert mass, and not circumducted as in organic hemiplegia. In the majority of cases hysterical hemiplegia was associated with complete hemianæsthesia. Hysterical hemiplegia might last an indefinite time and continue of the same flaccid character throughout. In hysterical hemiplegia the deep reflexes were not necessarily altered, and true ankle clonus was rare, whereas in organic hemiplegia the deep reflexes were exaggerated and ankle clonus was the rule. Roth,[6] in discussing the same problem, said that in organic hemiplegia aphasia in any of its forms might be present, and was not difficult to distinguish from hysterical mutism. Facial paralysis was excessively rare in hysterical hemiplegia: what was generally seen was a pseudo-paralysis, a hypotonus united to a hypertonus, with characteristic little jerking movements of the muscles of the opposite side of the face. In hysteria if deviation of the tongue occurred it presented itself in a variety of well-known capricious forms (hemispasm, systemic paresis, deviation to the opposite side). If hemianæsthesia occurred in organic hemiplegia it was less marked than paralysis, and the degree of anæsthesia was not necessarily the same over the entire half of the body, but there were none of the abrupt and capricious transitions seen in hysteria. The existence of an extensor type of plantar reflex was characteristic of organic disease.

**The Argyll-Robertson Pupil.**—Harris[7] says that loss of the pupil contraction to light may be looked upon as an almost certain sign of antecedent syphilis, congenital or acquired, and it is therefore to be met with not infrequently associated with tabes or general paralysis in syphilitic subjects who may or may not show other evidence of luetic infection. Harris has never yet seen it in Friedreich's disease or in disseminated sclerosis, and in only one instance in which a history of syphilis seemed to be fairly excluded—a man who suffered from spastic paraplegia with simple optic atrophy and some mental dulness. An Argyll-Robertson pupil may be either large or small, and in the latter power of contraction on convergence is usually lost or only slight, while further dilatation may be produced by skin stimulation. The very small pupils often met with cannot be explained simply as paralytic myosis, since the pupil is smaller than ever met with in sympathetic paralysis. The small size is probably due to contracture of the sphincter muscle, as very little dilatation occurs under atropine. The loss of the light reflex may be quite unilateral, though more often it is impaired on both sides. In such cases it will be found that if the sound pupil be shaded, it will contract when light is focussed on the affected pupil from any direction. This proves that the afferent path of the optic nerve is intact. Now the efferent path, and therefore the third nerve nucleus, is intact, as shown by the contraction of the pupil on accommodation. The lesion may therefore be concluded to affect fibres passing from the anterior corpora quadrigemina to the third nucleus. In birds and animals, where decussation of the optic nerves is complete, such fibres must decussate completely, for if light be thrown into one eye only that pupil contracts. In man, where decussation of the optic nerves is incomplete, and both pupils contract if light be thrown into one eye, there must be a semi-decussation of the fibres between the corpora quadrigemina and the third nuclei.

**Permanent Non-progressive Ataxia.** — Sanger Brown[8] reports three cases of this affection. All commenced with an acute illness, probably infective in nature, and in two associated with severe headache. This was followed by general muscular ataxia, conspicuously affecting the muscles of articulation. The ataxy once established became stationary, and the general health and mental condition were thenceforth normal. The speech condition is remarkably unlike that in any common nervous disease, the patient with mental powers unimpaired being reduced to a few monosyllabic grunts, or at best an occasional isolated simple word in which the dental and labial qualities are strikingly deficient. The localisation and pathology of the disease are as yet quite uncertain, but Brown believes it to be due to a toxin affecting the cerebellum.

**Adiposis Dolorosa.** — Descum[9] has described the autopsy on a case of adiposis dolorosa—a case, the first recorded, which he had published in 1888. Adiposis dolorosa is a disease characterised by the formation of fatty nodules throughout the body, causing great enlargement, and this is accompanied by severe pain, shooting and burning in character. In this particular case the arms were the first part to enlarge, and the shoulders, back, and sides of the chest were afterwards affected—the abdomen, hips, thighs, and legs immediately below the knees. The face, hands, feet, lower parts of the legs, and forearms were free from enlargement until the last. On numerous occasions excessive exacerbations of the pains occurred, and not infrequently a simultaneous increase of the swelling would be noted. She eventually died from fatty degeneration of the heart. At the autopsy an excessive deposit of fat was found in the subcutaneous tissue; the heart muscle was friable and yellowish; there was much subpericardial circumrenal fat. The omentum was filled with fat. On microscopical examination the fatty tissue was found to be indistinguishable from ordinary fat, but the peripheral nerves found in it presented undoubted evidence of peripheral neuritis, with a marked proliferation of the perineurium and endoneurium. There was slight degeneration of the columns of Goll in the spinal cord. The brain and pituitary body were normal. The thyroid gland presented marked changes: it was quite small in size. Large acini filled with colloid masses, smaller ones showing papillary outgrowths, and masses of secreting cells were found. These changes are in part indicative of hypertrophy. The explanation of this is doubtful: it might possibly be a hypertrophy of secreting tissue, a direct outcome of a general atrophy of the gland. In this connection it is interesting to note that adiposis dolorosa resembles myxœdema in presenting diminished sweating and occasional slowness of speech and mental irritability.

Another case of adiposis dolorosa, with autopsy, has been described by Burr.[10] The patient, aged 36 years, was very fat, weighing probably 300 pounds; but in addition to the general obesity there were large pendulous masses on the upper arms, thighs, and abdomen. She was admitted to hospital in a condition of extreme hebetude, and died with œdema of the lungs and Bright's disease. At the necropsy a tumour of the pituitary body, causing internal hydrocephalus, was found. The thyroid was normal in size and the ovaries small and hard. On microscopical examination the smaller nerve branches were found to show a high grade of interstitial neuritis. In the thyroid colloid degeneration with atrophy and absence of the secreting cells in many acini was found. There were many areas of small round cell infiltration and indications of an active inflammatory process. Thus the findings are the same as in Dercum's case: in both there was disease of the thyroid gland and of the finer nerve filaments. But the opinion that disease of the thyroid gland may cause adiposis dolorosa rests rather on analogy than proof. In Burr's case the tumour of the pituitary body was probably a coincidence, for in no other case of adiposis dolorosa have there been symptoms of interference with the optic chiasma or general symptoms of a brain tumour.

[5] Brit. Med. Jour., Aug. 18, 1900. [6] Ibid., Aug. 18, 1900. [7] Ibid., Sept. 29, 1900. [8] Amer. Jour. Med. Sci., June 1900. [9] Jour. of Nerv. and Ment. Dis., Aug. 1900. [10] Ibid., Oct. 1900,

